# An aluminium mold for intraoperative production of antibiotic-loaded PMMA knee prostheses

**DOI:** 10.3109/17453670902876771

**Published:** 2009-06-01

**Authors:** Sandro Kohl, Andreas Krueger, Christoph Roeder, Maximilian Hartel, Hendrik Kohlhof, Claudia Schneider, Stefan Eggli

**Affiliations:** ^1^Robert Mathys Foundation, BettlachSwitzerland; ^2^Department of Orthopaedic Surgery Inselspital, University of BernSwitzerland

## Introduction

For treatment of total knee prosthesis infections, a two-stage protocol with explantation of the infected components, antibiotic cement spacer implantation, and secondary reimplantation of the prosthesis after curing the infection is the accepted standard procedure (Wilde and Ruth 1988, Pitto and Spika 2004). The cement spacer is antibiotic-loaded, maintains the articular distance between femur and tibia, stabilizes the knee, and allows passive motion of the knee (Scott et al. 1993). Different techniques have been used for fabrication of this spacer, including intraoperative molding. The results of handcrafted spacers are unsatisfactory because such spacers have a rough surface and are not congruent. As a result, instability, limited range of motion, and excessive wear have been reported (Goksan and Freeman 1992, Hanssen et al. 1994).

We have developed a tapered aluminium mold for production of a custom made PMMA spacer like prostheses (CLSP) during the intervention. We consider the use of the CSLP as a promising approach for the treatment of complicated deep TKA infections since this technique is time sparing, cheap and easy to apply in all surgical theatres.

### Construction of the spacer mold

**Figure 1.**
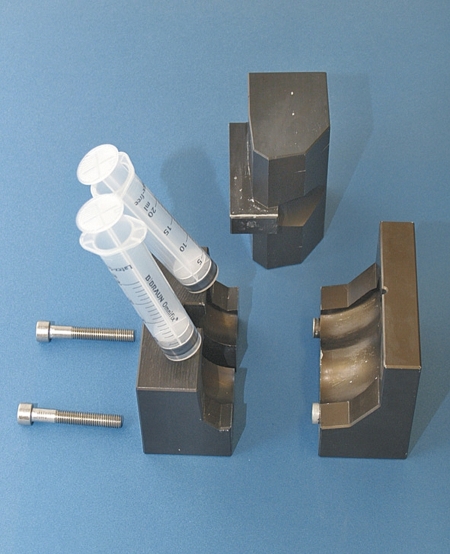
Femoral mold, composed of 3 parts. It can be filled through 2 channels with tubes.

**Figure 2.**
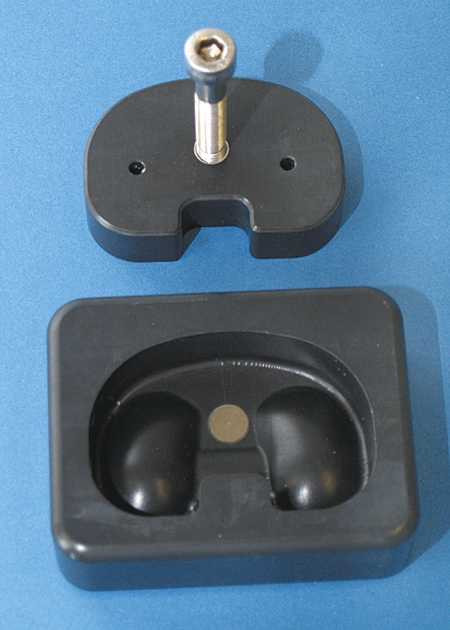
The 2 parts of the tibial mold.

The 2 molds were produced in a computerized numerical-control sinking machine (DMU 70eV-process) based on the design of the balanSys knee system (size B; D) (Mathys AG, Bettlach, Switzerland). The mold for the femoral spacer consists of 3 parts and for the tibial spacer, of 2. It is made of high-strength aluminium (AA7075 T6 3.4365; density 2.8 kg/dm^3^ and tensile strength 520–560 Nm/mm^2^) and coated with Altef. In this process, the surface of the base material is converted into a ceramic coating in which Teflon is integrated. Half of the coating penetrates into the base material, which results in an increase of 25 µm, leading to a standard coating thickness of 50 µm. The mold can be cleaned and sterilized for re-use (Figure 1 and 2).

### Surgical technique and postoperative protocol

The first step includes explantation of the prosthetic components and removal of the cement mantle. An extensive debridement of the infected joint is performed. Biopsies are taken for microbiological culture and histology. The appropriate size for the components is assessed on conventional AP and lateral knee radiographs. The parts of the femoral mold component are mounted with 2 screws. If necessary, special antibiotics can be added to the cement, based on the cultures and antibiogram. The mold is filled through 2 channels on the upper side, using a 20-mL tube. After polymerization of the cement, the screws, which interlink the mold, are opened and the femoral spacer component can be easily removed from the mold. Since the cement expands during polymerization, pressure inside the closed mold rises and creates a smooth surface on the final cement spacer. The femoral component is implanted first with a small portion of additional cement.

**Figure 3.**
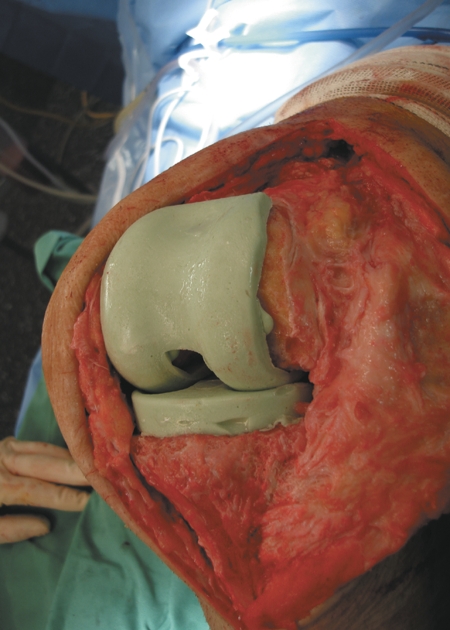
Implantation of the intraoperative molded PMMA knee spacer.

**Figure 4.**
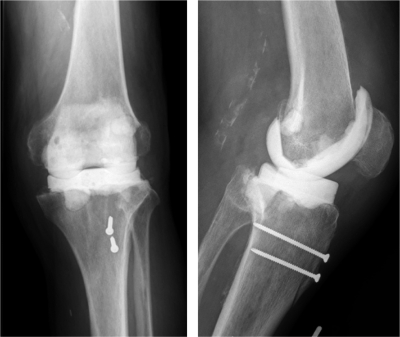
After implantation of the PMMA spacer.

Then the distance between the tibia and the femur is measured in neutral position. The tibial mold is filled with PMMA cement according to the distance measured. After polymerization of the cement, the tibial part of the spacer is removed from the mold and the mounting cement on the posterior and lateral side of the spacer is removed with a Luer pincer. The tibial component is then implanted with a small portion of cement (Figure 3). The stability and range of motion is tested, and then the wound can be closed (Figures 4).

Depending on the agent and the intraoperative findings, an individual protocol of intravenous and oral antibiotics is indicated. Continuous passive motion is started 24 h postoperatively, with no limitations. Ambulation is started (with two crutches and partial weight bearing of 20 kg) on the third day after surgery.

## Discussion

The two-stage protocol for revision of infected TKA was proposed by Insall et al. (1983) and has been widely adopted since then (Wilde and Ruth 1988, Masri et al. 1994, Hofmann et al. 1995). However, the interposition of a PMMA spacer is still considered to be controversial (Zimmerli et al. 2004). A static cement spacer often induces significant arthrofibrosis with stiffness of the joint, and can cause bone loss because of instability (Windsor et al. 1990, Fehring et al. 2000).

To date, intraoperative manually produced articulating spacers are the most commonly used (Nazarian et al. 2003, Villanueva et al. 2006). Such spacers often show insufficient congruency and have a rough surface, which results in instability and excessive wear. Thus, thick scarring of the joint capsule with limited motion is often found at reimplantation, which requires extensive debridement and arthrolysis. The only commercially available molding system is the StageOne Knee Cement Spacer Molds (Biomet Orthopaedics Inc., Warsaw, IN). This produces significantly better congruency of the articulating parts, with better stability (Castelli et al. 2002). Because the molds are made of silicone, the surface of the spacers is not compressed during the hardening process. Also, these molds are only for single use, which is quite costly.

We have developed an Altef tapered aluminium mold for production of a custom-made PMMA spacer during intervention. The stable mechanical construction of the mold results in a high densification of the cement during the oxygenic hardening process. Stable implantation of the spacer is achieved with a second cementation. In this way, a low-friction articulation with excellent stability and a range of motion from 0–120 degrees is achieved.

The main advantages of this system are the stability of the knee after implantation because of the high congruency of the spacer components, and low friction between the articulating surfaces. Thus, almost no PMMA wear particles can be found at reimplantation of the prosthesis. Stability in combination with a low-friction coefficient permits immediate motion training, which results in a substantially better range of motion at reimplantation of a new implant. Other positive attributes include the possibility of adding high doses of antibiotics and the possibility of correct reconstruction of the joint line and the anatomical axis of the leg. Finally, the reusability of the system is cost-efficient.
